# Influence of a Tribally Led Children's Environmental Education Program on Shellfish Harvesting Beliefs and Behavioral Intentions

**DOI:** 10.1029/2025CSJ000128

**Published:** 2025-09-03

**Authors:** Hugh B. Roland, Jacob Kohlhoff, Travis R. Moore, Kari Lanphier, Lindsey Pierce, Julian Narvaez, Aissa Yazzie, Christopher Whitehead, Jeff Feldpausch, Matthew O. Gribble

**Affiliations:** ^1^ Department of Health Policy and Organization University of Alabama at Birmingham School of Public Health Birmingham AL USA; ^2^ Division of Occupational, Environmental, and Climate Medicine Department of Medicine University of California, San Francisco San Francisco CA USA; ^3^ Friedman School of Nutrition Science and Policy Tufts University Boston MA USA; ^4^ Sitka Tribe of Alaska Sitka AK USA; ^5^ Division of Native Land and Resources Central Council of the Tlingit and Haida Indian Tribes of Alaska Juneau AK USA; ^6^ Hoonah Indian Association Hoonah AK USA; ^7^ Northwest Indian College Bellingham WA USA

**Keywords:** adaptive behavior, community health education, adolescent psychology, shellfish poisoning, Alaska

## Abstract

To increase the understanding of shellfish toxin risks and support safe harvesting practices, the Sitka Tribe of Alaska develops and organizes environmental education programs. This study (ClinicalTrials.gov ID: NCT05247229) evaluates the Tribe's new middle school program, drawing on the theory of planned behavior to investigate pre‐post‐program shifts in beliefs and behavioral intentions related to shellfish harvesting and mitigating exposure risks by checking a tribally run website with near real‐time toxin level data. Participants included 50 middle school students across three Southeast Alaska communities—Sitka, Hoonah, and Juneau. Research activities included pre‐ and post‐program surveys and interviews. We used generalized estimating equation linear regression of survey data to investigate pre‐post‐program changes in beliefs and behavioral intentions related to shellfish harvesting and risk reduction and how changes in beliefs relate to changes in behavioral intentions. Interviews contextualized beliefs and behavioral intentions measured in surveys. Following the program, participants reported more positive perceptions and increased behavioral intentions related to shellfish harvesting and checking toxin levels, although differences emerged across sites and Alaska Native identity. Participants' understanding of the risk reduction strategy and confidence in abilities to check toxin levels also increased, suggesting that integrating risk perception in the theory of planned behavior and practical risk reduction strategies in environmental education tailored to local ecological and cultural contexts can be effective in promoting safe behaviors. Additionally, participants emphasized the influence of their family's harvesting practices on their beliefs and behaviors, suggesting the importance of family engagement in environmental education.

## Plain Language Summary

This study assesses the impact of a children's education program aimed at increasing understanding of shellfish toxin risks and promoting safe shellfish harvesting. Drawing on a psychological theory called the theory of planned behavior that connects beliefs to intended behaviors, we evaluated shifts in beliefs and behavioral intentions related to shellfish harvesting and pursuing an accessible toxin exposure risk reduction strategy. The program was implemented in three middle schools across three Southeast Alaska communities and the related mixed methods research included surveys and interviews with middle school students in the program. Overall, participants reported more positive perceptions and greater behavioral intentions toward shellfish harvesting and risk reduction, although differences emerged between sites and participants' identity as Alaska Native or non‐Native. Our findings suggest that providing an accessible risk reduction strategy as part of environmental education tailored to local environmental and cultural contexts can be effective in promoting safe environmental and health behaviors. Our findings also emphasize the influence of family shellfish harvesting practices on perceptions and behaviors, suggesting the importance of family engagement in environmental education.

## Key Points

- This study assessed the impact of a children's education program on understanding of shellfish toxin risks and safe harvesting strategies
- Pre‐ and post‐program surveys and interviews indicated increased behavioral intentions to check toxin levels and harvest safely
- Findings emphasized the effectiveness of education that integrates accessible risk reduction within local ecological and cultural contexts

## 1 Introduction

### 1.1 Background: Shellfish Toxin Exposures in Southeast Alaska

Shellfish are a culturally important food for the Tlingit, Haida, and Tsimshian people living in their traditional territories in present‐day Southeast Alaska. Shellfish harvesting appears as a motif in traditional Alaska Native artwork (Thorsen & Knapp, 2008, p. 27), is included in traditional food education (Neumann, 2013), and is widely practiced in Southeast Alaska (Ibarra, 2021). However, consuming shellfish that have not been tested for shellfish toxins has severe health risks, particularly paralytic shellfish poisoning (PSP), which can be fatal. Since 1973, there have been over 200 recorded cases of PSP in Alaska, with many more likely unreported (Harley et al., 2020). Owing to harvesting traditions and culturally central consumption among Tlingit, Haida, Tsimshian, and other coastal Alaska Native people, Alaska Natives have greater risks of shellfish toxin exposure and PSP compared to non‐Natives (Gessner & Schloss, 1996; Harley et al., 2020). Other shellfish toxins pose further risks and may contribute to additional health disparities, such as higher colorectal cancer rates among Alaska Natives than non‐Natives (Cordier et al., 2000; Haverkamp et al., 2023).

Harvesters have used traditional knowledge to lower toxin exposure risks, such as only harvesting during certain months (Roland, Kohlhoff, Lanphier, Hoysala, et al., 2024), but climate change‐related processes that alter the frequency, magnitude, and geographic distribution of the harmful algal blooms (HABs) that produce high toxin levels in shellfish decrease the predictability of exposures and the reliability of traditional knowledge (Anderson et al., 2021; Yan et al., 2020). Traditional knowledge, also called Indigenous knowledge (Bruchac, 2020), is frequently defined as a cumulative body of knowledge, practices, and beliefs handed down across generations (Berkes et al., 2000; Gómez‐Baggethun, 2022; Semali et al., 2002; Toledo, 2002). Traditional knowledge is often associated with place and the ways in which longtime residents have come to understand themselves in relation to a place (Semali et al., 2002), but local knowledge is not traditional knowledge if knowledge of a place is not associated with the experiences of Indigenous Peoples (Saami Council, 2024).

To reduce exposure risks, a tribally led toxin testing network called Southeast Alaska Tribal Ocean Research (SEATOR) was created in 2014. Tribal partners in the SEATOR network regularly collect shellfish samples from community harvesting sites across Southeast Alaska, encourage community members to send their own samples for testing, and share toxin data to inform harvesting decisions. SEATOR maintains a website where near real‐time toxin levels are posted, and checking toxin levels on this website is a primary adaptation strategy for shellfish harvesters in the region (Harley et al., 2020).

### 1.2 Research Questions: Influence of the Theory of Planned Behavior on Children's Behavioral Intentions

Education is critical to increase awareness and understanding of shellfish toxin risks and risk reduction strategies in Southeast Alaska (Roland, Kohlhoff, Lanphier, Hoysala, et al., 2024). In this clinical trial (ClinicalTrials.gov ID: NCT05247229), we assess the influence of a new middle school education program designed to promote awareness of shellfish toxin risks, harvesting practices, and toxin exposure risk reduction strategies to support the safe continuation of this culturally important practice. Specifically, we evaluate the explanatory value of the theory of planned behavior (TPB) in understanding changes in children's perceptions and behavioral intentions related to shellfish harvesting and toxin exposure risk reduction. The TPB is a behavioral science framework used to understand factors influencing individuals' intentions to engage in actions (Godin & Kok, 1996). The TPB identifies three types of beliefs: behavioral beliefs (attitudes toward the behavior), normative beliefs (perceived attitudes of peers and respected figures toward the behavior), and control beliefs (perceived ability to perform the behavior) (Ajzen, 1991). According to the TPB, when these beliefs align, there is a higher likelihood that a person will intend to perform a behavior.

Related to the TPB, we investigate two questions. First, we evaluate pre‐post‐program changes in TPB beliefs and behavioral intentions related to shellfish harvesting and consumption and checking the SEATOR website for toxin levels. This was the focus of the clinical trial. Second, we examine how changes in TPB beliefs relate to changes in behavioral intentions. This area of scrutiny was not part of the clinical trial but contributes to our understanding of pre‐post‐program changes. In both analyses, we investigate differences across the three participating communities (Sitka, Hoonah, and Juneau), between Alaska Native and non‐Native identifying participants, and across gender identity (girls, boys, and nonbinary).

### 1.3 Literature Review

#### 1.3.1 Environmental Health Literacy (EHL) and Ocean Literacy

EHL encompasses the personal, cognitive, and social skills necessary to access, understand, and use health‐related information to maintain and improve well‐being (Finn & O’Fallon, 2017; A. G. Hoover, 2019; Nutbeam, 2000). Ocean literacy extends the principles of EHL to emphasize the vital role of the ocean in human health and the environment (Intergovernmental Oceanographic Commission, 2021; McKinley et al., 2023). In Southeast Alaska, where Alaska Native communities depend on the ocean for food, cultural practices, and economic activities, ocean literacy may foster an understanding of marine ecosystems, promote collective responsibility, and empower community‐led solutions.

Despite advancements in health literacy, interventions relying mainly on communication and education often fail to produce behavior changes or address disparities in health outcomes (Nutbeam, 2000). This is particularly true in Indigenous communities, where colonial legacies and systemic inequities have created more environmental health risks (Czyzewski, 2011; E. Hoover et al., 2012). In Indigenous communities, EHL and ocean literacy frameworks may be most effective when they embrace Indigenous cultural values and traditional knowledge (McKinley et al., 2023). This means recognizing that EHL and ocean literacy are relatively new terms for processes that Indigenous Peoples have practiced for millennia (Worm et al., 2021) and designing interventions that resonate with collective values, address systemic inequities, and empower individuals to act as stewards of their environment (Simonds et al., 2019). For example, efforts to balance health risks and benefits of traditional foods have tailored interventions to cultural and environmental contexts (Dellinger et al., 2019, 2022; Laird et al., 2013), and health‐supporting interventions emphasizing language, culture, and collectivism have demonstrated how reconnecting to traditional food practices can mitigate intergenerational trauma, promote well‐being, and foster self‐determination (Hilgendorf et al., 2019). Further, EHL in Indigenous communities may enhance abilities to recognize hazards, advocate for environmental justice, and protect residents' health by offering clear, actionable information and making scientific, legal, and regulatory information more accessible (Simonds et al., 2019).

#### 1.3.2 The Theory of Planned Behavior With Children and in Indigenous and Public Health Settings

In public health initiatives, the TPB has been used to shift behavioral intentions by addressing psychological and social determinants of health behaviors. Public health efforts have facilitated positive shifts in dietary choices and physical activity by focusing on attitudes, subjective norms, and perceived behavioral control (Ajzen, 1991; Conner & Armitage, 1998; Sommer & Kouba, 2015; Stranieri et al., 2016). Additionally, interventions have supported attitude and perception modification by emphasizing community norms and using hands‐on, place‐based learning (Zimmerman & Weible, 2017). In the context of environmental health initiatives, expanding the TPB to include worry and risk perception can enhance understanding and adoption of health‐supporting behaviors (Brewer et al., 2007; Rimal & Real, 2003; Schmiege et al., 2009). Risk perceptions affect perceived behavioral control by influencing individuals' views on the ease or difficulty of carrying out risk reduction strategies (Rimal & Real, 2003). Targeting specific risk perceptions (e.g., deliberative, affective, and experiential) and efficacy beliefs relevant to audience characteristics and contexts may further increase impact (Ferrer & Klein, 2015; Loewenstein et al., 2001; Shi & Kim, 2020; Slovic et al., 2013).

TPB‐based interventions targeting children have identified TPB‐related strategies particularly influential in contributing to children's behavior change, including encouraging positive attitudes toward healthy behaviors, reinforcing supportive social norms, and increasing perceived behavioral control (De Leeuw et al., 2015; Fishbein & Ajzen, 2011; McEachan et al., 2011). Research also suggests that integrating empathic concern and moral norms into environmental and TPB‐based interventions can increase effectiveness by aligning programming with children's personal values and community responsibilities (Bamberg & Möser, 2007; De Leeuw et al., 2015; Steg & Vlek, 2009). TPB‐based interventions targeting Indigenous audiences have influenced behaviors related to traditional foods and cultural practices (Burnette et al., 2020; Fila & Smith, 2006; Redwood et al., 2008) and have been particularly successful in promoting health and cultural continuity by using community‐defined approaches (Blue Bird Jernigan et al., 2012; Wexler et al., 2014).

From this literature on the TPB with children and in Indigenous and public health settings, several factors within the TPB framework emerge as particularly influential in driving behavior change: perceived behavioral control, subjective norms and cultural relevance, and contextual factors such as cultural beliefs and barriers to behaviors. These factors demonstrate that although the TPB's core constructs are broadly applicable, their effectiveness may be enhanced by considering contextual and cultural characteristics. These insights align with broader calls to address context and place in environmental health research and practice (Cordner et al., 2016) and were integrated into the design of the education program evaluated in this study. Reflecting the importance of tailored interventions, the education program was designed for middle school‐aged students (ages 11–13) and the Southeast Alaska ecological and cultural context. Reflecting the role of perceived risks in influencing behavior change, the program integrates risk perception within the TPB by targeting control beliefs, promoting a specific risk reduction strategy, and supporting self‐efficacy in shellfish harvesting decision‐making.

## 2 Data and Methods

### 2.1 Research Team and Reflexivity

This study was a partnership between researchers, educators, and environmental protection staff at the University of Alabama at Birmingham (UAB), the University of California, San Francisco (UCSF), and the Sitka Tribe of Alaska (STA). The community‐academic partnership was initiated in 2016 when academic partners were invited to support the efforts of a tribe‐school district partnership in Sitka and a tribally led environmental monitoring program. The partnership involves shellfish toxin testing and modeling and K‐12 educational programming to promote cultural, environmental, and health literacy and increase awareness of shellfish poisoning risk reduction strategies and STA risk reduction resources. The intervention evaluated in this study is a new education program for middle school students. This work is community co‐led and participatory (Key et al., 2019), reflects reflexive research ethics principles (Cordner et al., 2016), and follows best practices for co‐created community engagement in oceans and human health research (Carson et al., 2022). Community‐based participatory research offers a model for fostering trust, reciprocity, and long‐term collaboration with Indigenous communities (Manson et al., 2004). The research instruments used in this study were developed collaboratively by members of the author team with academic and STA affiliations. Surveys were conducted by coauthors implementing the education program in Sitka, Hoonah, and Juneau and respectively affiliated with STA, the Hoonah Indian Association, and the Central Council of the Tlingit and Haida Indian Tribes of Alaska. Interviews, across communities, were conducted by coauthors with STA and UAB affiliations. This research received human subjects research approval from the University of Alabama at Birmingham Institutional Review Board (IRB protocol number 300006786). In reporting, we draw on three guidelines, including criteria specific to health research with Indigenous Peoples (Huria et al., 2019), environmental education research (Smith‐Sebasto, 2000), and group‐based behavior‐change interventions (Borek et al., 2015). We present consolidated criteria for strengthening reporting of health research involving Indigenous Peoples in Appendix A (Huria et al., 2019) and relevant items from the other two guidelines in this methods section.

### 2.2 Middle School Education Program

The middle school education program that this study evaluates teaches participants about traditional knowledge and shellfish harvesting practices in Southeast Alaska, risks posed by shellfish toxins, and risk reduction strategies. The program was designed by STA, run by education and environmental coordinators working for STA, the Hoonah Indian Association, and the Central Council of the Tlingit and Haida Indian Tribes of Alaska, and featured guest speakers, such as staff scientists responsible for shellfish toxin testing and tribal members with expertise in local knowledge and traditional knowledge (Roland, Kohlhoff, Lanphier, Yazzie, et al., 2024). The program was initially implemented only in a middle school in Sitka and was expanded to schools in Hoonah and Juneau in response to Tribes' interest. The semester‐long, 1‐hr program took place weekly, either as an after‐school program or during school hours as a non‐graded elective, depending on the structure most feasible for schools. Lessons offered participants hands‐on learning such as experience with laboratory techniques, microscopy, plankton tows, and shellfish harvesting. The program was divided into two units: the first focused on HABs and PSP and the second focused on intertidal zones and shellfish harvesting. Research activities included pre‐ and post‐program interviews and surveys and a follow‐up survey 1 year after participation. Pre‐ and post‐program surveys were the primary outcome measure of the clinical trial, and pre‐ and post‐program interviews and the 1 year post‐program survey were secondary measures.

The program was designed with the TPB in mind and especially targeted control beliefs. The program aimed to influence behavioral beliefs by educating students about shellfish harvesting through hands‐on, place‐based learning; normative beliefs by emphasizing that harvesting with parents or guardians is customary; control beliefs by highlighting toxin exposure risk reduction strategies and tribal PSP prevention resources; and, ultimately, behavioral intentions through shifting behavioral, normative, and control beliefs.

### 2.3 Sample and Consent

Study participants were recruited from all middle school students (grades 6–8) in Blatchley Middle School in Sitka, Hoonah City School in Hoonah, and Dzantik'i Heeni Middle School in Juneau. The program enrolled 11 participants in Sitka, 20 in Hoonah, and 19 in Juneau for a total of 50 participants across communities. Research participation was not required to participate in the education program, and students opted into the survey and interview research components through a tiered informed consent process. Written consent was obtained from parents after information on research activities was shared, and verbal consent was obtained from participants before data collection activities. Of the 50 study participants, all consented to participate in the surveys and all but one consented to participate in the interviews. Participants in either or both research components were offered a $10 cash card incentive for each lesson attended, with a total possible incentive of $100 for attending all 10 lessons. Incentives were used to encourage program participation and regular attendance.

### 2.4 Survey Design

A five‐point (1–5) Likert scale survey was administered to participants at the beginning and end of the program, as well as 1 year after program completion. The instrument used in these three instances of data collection included 16 questions relevant to the three types of TPB beliefs—behavioral beliefs (attitudes toward the behavior), normative beliefs (perceived attitudes of peers and respected figures toward the behavior), and control beliefs (perceived ability to perform the behavior)—and was divided into two sections. In the first section, on shellfish harvesting, questions asked about participants' perceptions of and experience with shellfish harvesting, perceptions of peers' views about shellfish harvesting, and sense of agency and behavioral intentions related to engaging in shellfish harvesting. For example, related to normative beliefs: “Most of the people who are important to me would approve of me participating in subsistence clam harvesting” (disagree—agree Likert scale). In the second section, on checking toxin levels using the SEATOR website, questions asked about participants' perceptions of this risk reduction behavior, perceptions of peers' views about checking toxin levels, and sense of agency and behavioral intentions related to checking toxin levels. For example, related to control beliefs: “I am confident that I can check the SEATOR website before clam harvesting” (disagree—agree Likert scale). In both sections, the last question asked about behavioral changes related to program learning and thus was relevant in only the post‐program survey. Full survey response data are presented in Appendix B.

### 2.5 Survey Data Analysis

#### 2.5.1 Accounting for Missing Data

Missing data at the person‐visit level were imputed for age (*n* missing = 6) and each of the questions (*n* missing = 9–20, depending on question), treating each person‐visit as a separate observation. The predictor variables in the imputed model included all question responses and all regression model predictors: age, gender identity, Alaska Native identity, and site. We used list‐wise deletion, excluding cases from analysis if data wrere missing, for gender (*n* missing = 10), Alaska Native identity (*n* missing = 6), and pre‐post‐program differences (*n* missing = 9–15, depending on question). No site data were missing. We generated 80 imputed data sets via multiple imputation by chained equations (Azur et al., 2011). Across our models, the largest average relative increase in variance (RVI) due to nonresponse was 0.44 and the largest fraction of missing information (FMI) was 0.55, suggesting that our analysis based on 80 imputed data sets was reasonable. Statistical analyses were conducted using STATA 18.0 M/P (StataCorp, 2023).

#### 2.5.2 Pre‐Post‐Program Changes in Theory of Planned Behavior Beliefs and Behavioral Intentions

To evaluate pre‐post‐program changes in beliefs and behavioral intentions related to shellfish harvesting, consumption, and risk reduction, we tested the association between pre‐ and post‐program responses (i.e., the intervention effect). We included covariates for site (Sitka, Hoonah, and Juneau), age, Alaska Native identity (Alaska Native, non‐Native), and gender identity (girls, boys, and nonbinary). To consider within person pre‐post‐program changes in TPB beliefs and behavioral intentions, we used generalized estimating equation linear regression (GEE) to cluster pre‐post measures (Liang & Zeger, 1986). We also evaluated differences in pre‐post‐program changes in TPB beliefs and behavioral intentions among the three participating communities and Alaska Native and gender identities using interacted models. Results of interacted models are presented in Appendix C.

We present individual participants' responses to the 1‐year post‐program survey as the number of participants in this follow‐up survey was small. When we closed the study on 31 July 2024, only seven participants had completed the program at least one year prior and were available for one year post‐program recontact, having completed the program in either the fall 2022 or spring 2023 semester. These participants were all from Sitka. Of these 7 participants, 4 completed the follow‐up survey. One‐year post‐program survey responses are presented in Appendix D.

#### 2.5.3 Relationships Between Changes in Theory of Planned Behavior Beliefs and Changes in Behavioral Intentions

To characterize TPB constructs most influential in shifting planned behaviors, we again used GEE to test the association between pre‐post‐program changes in TPB beliefs and pre‐post‐program changes in harvesting and risk reduction behavioral intentions. Similar to the models we used to assess pre‐post‐program changes, we adjusted for site, age, Alaska Native identity, and gender identity. We also adjusted for changes in other TPB beliefs related to either harvesting or risk reduction, depending on the behavioral intention outcome.

### 2.6 Interview Structure

Together with the surveys, pre‐ and post‐program structured interviews followed a convergent parallel study design, with interviews contextualizing the beliefs and behavioral intentions measured in surveys. For example, interview questions related to normative beliefs asked: “How do the people in your life (friends, family, etc.) feel about subsistence clam harvesting? What do you think of their feelings toward subsistence clam harvesting? Have these individuals' feelings about subsistence clam harvesting influenced how you feel about harvesting? If so, how?” Similar to the survey, interviews were organized by TPB constructs. The 10 question interview script is included as Appendix E.

### 2.7 Interview Data Analysis

Interview audio recordings were transcribed using Otter.ai transcription software. Coauthors who conducted interviews reviewed these initial transcripts for quality control, listening to each recording and editing transcripts for accuracy. A codebook was generated using both inductive and deductive approaches. First, codes were identified based on major themes in the interview protocol and relevant to the TPB. Next, all transcripts were reviewed to identify further themes not captured in deductively developed codes. This grounded theory approach was important to identify new themes raised by participants. The inductive coding process was open and collaborative, with members of the research team involved in the process meeting frequently during coding scheme generation and coding. Each interview was coded by a minimum of two research team members. To support high intercoder reliability, team members checked intercoder agreement and collaboratively reviewed coding results throughout the coding process, identifying differently coded excerpts and reaching consensus on correct coding. Data were coded and analyzed using Dedoose software (Dedoose Version 9.2.12, 2021).

## 3 Results

### 3.1 Survey Results

#### 3.1.1 Pre‐Post‐Program Changes in Theory of Planned Behavior Beliefs and Behavioral Intentions

**Figure 1:**
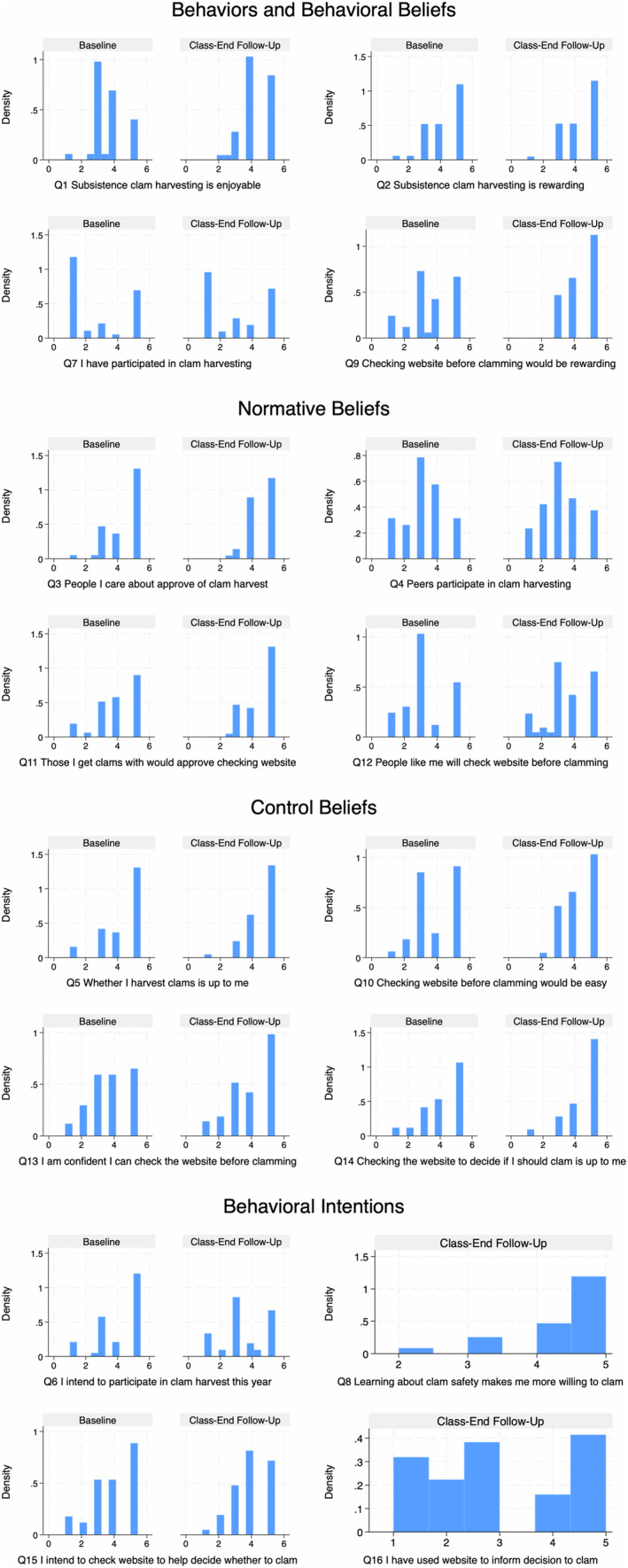
Survey response distributions by theory of planned behavior construct.

Across sites and demographic groups, the most agreed with statement pre‐program reflects normative beliefs that people important to participants would approve of their participation in clam harvesting (Q3). Other questions that received strong agreement pre‐program reflect the other TPB beliefs. Related to control beliefs, participants agreed that participating in clam harvesting was up to them (Q5). Related to behavioral beliefs, participants agreed that clam harvesting was rewarding (Q2). The most agreed with questions post‐program included these questions with strong pre‐program agreement in addition to questions spanning TPB constructs about checking the SEATOR website for toxin levels. Related to behavioral beliefs, participants agreed that checking the SEATOR website would be rewarding (Q9). Related to normative beliefs, participants agreed that the people they harvest with would approve of them checking the SEATOR website (Q11). Related to control beliefs, participants agreed that they would be able to check the SEATOR website (Q14). Post‐program responses also reflect the intervention's success in promoting safe shellfish harvesting. Related to planned behaviors, participants mostly agreed that since learning about clam harvest safety, they felt more willing to harvest (Q8) (See Figure 1, Table 1).

**Table 1:** Differences in Theory of Planned Behavior Beliefs and Behavioral Intentions Between Pre‐ and Post‐Program Surveys From Generalized Estimating Equation Linear Regressions Accounting for Clustering of Longitudinal Observations Within Person, Adjusted Associations and 95% Confidence Intervals: Overall Association (Results for Site‐, Alaska Native Identity‐, and Gender Identity‐Interacted Models in Appendix C)

	Overall association (95% CI)
	*p*‐value (*X* ^2^ test)
Q1 (Behavioral beliefs): Subsistence clam harvesting is enjoyable	0.51 (0.16, 0.86)
	*p* = 0.01
Q2 (Behavioral beliefs): Subsistence clam harvesting is rewarding	0.09 (−0.34, 0.52)
	*p* = 0.67
Q3 (Normative beliefs): Most of the people who are important to me would approve of me participating in subsistence clam harvesting	0.13 (−0.15, 0.41)
	*p* = 0.35
Q4 (Normative beliefs): Most of my peers (classmates, friends, etc.) participate in subsistence clam harvesting	0.09 (−0.43, 0.61)
	*p* = 0.73
Q5 (Control beliefs): Whether or not I participate in subsistence clam harvesting is up to me	0.20 (−0.13, 0.52)
	*p* = 0.23
Q6 (Behavioral intention): I intend to participate in subsistence clam harvesting this year	−0.42 (−0.96, 0.11)
	*p* = 0.12
Q7 (Behavior): I have participated in subsistence clam harvesting	0.41 (−0.23, 1.05)
	*p* = 0.21
Q9 (Behavioral beliefs): Checking the SEATOR website before clam harvesting would be rewarding	0.71 (0.27, 1.15)
	*p* = 0.00
Q10 (Control beliefs): Checking the SEATOR website before clam harvesting would be easy	0.37 (0.00, 0.73)
	*p* = 0.05
Q11 (Normative beliefs): The people who I go clam harvesting with would approve of me checking the SEATOR website before clam harvesting	0.37 (−0.07, 0.82)
	*p* = 0.10
Q12 (Normative beliefs): Most people like me (classmates, friends, etc.) will check the SEATOR website before clam harvesting	0.30 (−0.30, 0.91)
	*p* = 0.32
Q13 (Control beliefs): I am confident that I can check the SEATOR website before clam harvesting	0.22 (−0.25, 0.70)
	*p* = 0.36
Q14 (Control beliefs): Checking the SEATOR website to decide if I should go clam harvesting is up to me	0.35 (−0.07, 0.78)
	*p* = 0.11
Q15 (Behavioral intention): I intend to check the SEATOR website to help decide if I should go clam harvesting	0.07 (−0.41, 0.54)
	*p* = 0.78
Overall association: Tests for the association between pre‐ and post‐program response means (i.e., the intervention effect), adjusting for site, age, Alaska Native identity, and gender identity	

Comparing pre‐ and post‐program survey responses, we find, in all but one question, greater post‐program agreement with survey statements, indicating more positive perceptions of and interest in harvesting and toxin risk reduction post‐program. The largest pre‐post‐program shifts were observed in behavioral belief questions, with greater post‐program participant agreement, on average, that subsistence clam harvesting is enjoyable (Q1) and that checking the SEATOR website before harvesting would be rewarding (Q9). Participant agreement post‐program also increased, on average, in other questions about checking the SEATOR website for toxin levels, including, related to control beliefs, that checking the SEATOR website would be easy (Q10).

#### 3.1.2 Relationships Between Changes in Theory of Planned Behavior Beliefs and Changes in Behavioral Intentions

To better understand if changes in particular TPB beliefs are more likely to drive changes in behavioral intentions, we investigated how pre‐post‐program changes in TPB beliefs related to changes in behavioral intentions. Beliefs corresponding to each TPB construct appear influential in shifting harvesting and risk reduction planned behaviors. Related to harvesting planned behaviors, greater post‐program agreement with the behavioral belief question, that harvesting is enjoyable (Q1), and the normative belief question, that people important to participants would approve of them harvesting (Q3), correspond with increases in intentions to harvest within the year (Q6). Highlighting the complexity of harvesting decision making, we observed one significant change in the opposite direction, where greater agreement with the behavioral belief question, that harvesting is rewarding (Q2), corresponds with decreases in intentions to harvest within the year (Q6). Related to risk reduction planned behaviors, greater agreement with the behavioral belief question, that checking the SEATOR website would be rewarding (Q9), and with the control belief question, that participants felt confident checking the SEATOR website (Q13), correspond with increases in intentions to check the SEATOR website to inform harvesting decisions (Q15) (See Table 2).

**Table 2:** Adjusted Associations Between Pre‐Post‐Program Differences in Theory of Planned Behavior Beliefs and Pre‐Post‐Program Differences in Behavioral Intentions From Generalized Estimating Equation Linear Regressions Accounting for Clustering of Longitudinal Observations Within Person, Adjusted Associations and 95% Confidence Intervals

	Association (95% CI)
	*p*‐value (*X* ^2^ test)
Behavioral intention: Q6: I intend to participate in subsistence clam harvesting this year	
Q1 (Behavioral beliefs): Subsistence clam harvesting is enjoyable	0.60 (0.23, 0.96)
	*p* = 0.00
Q2 (Behavioral beliefs): Subsistence clam harvesting is rewarding	−0.33 (−0.62, −0.04)
	*p* = 0.02
Q3 (Normative beliefs): Most of the people who are important to me would approve of me participating in subsistence clam harvesting	0.33 (0.01, 0.65)
	*p* = 0.05
Q4 (Normative beliefs): Most of my peers (classmates, friends, etc.) participate in subsistence clam harvesting	0.21 (−0.04, 0.46)
	*p* = 0.09
Q5 (Control beliefs): Whether or not I participate in subsistence clam harvesting is up to me	0.13 (−0.16, 0.41)
	*p* = 0.38
Q7 (Behavior): I have participated in subsistence clam harvesting	0.09 (−0.08, 0.27)
	*p* = 0.29
Behavioral intention: Q15: I intend to check the SEATOR website to help decide if I should go clam harvesting	
Q9 (Behavioral beliefs): Checking the SEATOR website before clam harvesting would be rewarding	0.29 (0.11, 0.47)
	*p* = 0.00
Q10 (Control beliefs): Checking the SEATOR website before clam harvesting would be easy	−0.02 (−0.21, 0.16)
	*p* = 0.79
Q11 (Normative beliefs): The people who I go clam harvesting with would approve of me checking the SEATOR website before clam harvesting	0.07 (−0.11, 0.25)
	*p* = 0.45
Q12 (Normative beliefs): Most people like me (classmates, friends, etc.) will check the SEATOR website before clam harvesting	0.05 (−0.09, 0.19)
	*p* = 0.50
Q13 (Control beliefs): I am confident that I can check the SEATOR website before clam harvesting	0.45 (0.28, 0.62)
	*p* = 0.00
Q14 (Control beliefs): Checking the SEATOR website to decide if I should go clam harvesting is up to me	0.14 (−0.03, 0.31)
	*p* = 0.11
Association: Tests for the association between mean pre‐post‐program differences in TPB beliefs and mean pre‐post‐program differences in behavioral intentions, adjusting for site, age, Alaska Native identity, gender identity, and changes in other TPB beliefs	

#### 3.1.3 Site Differences

Among sites, the largest differences that emerged were between participants in Hoonah and participants in Sitka and Juneau. Pre‐program, participants in Hoonah reported more positive behavioral beliefs related to perceptions of shellfish harvesting (Q2), as well as greater participation (Q7) and peer participation (Q4) in harvesting than participants in Sitka or Juneau. We did not observe statistically significant differences in pre‐post‐program changes in TPB beliefs and behavioral intentions between communities. In the post‐program survey, pre‐program community differences mostly persisted. Post‐program, participants in Hoonah again reported more frequent peer participation in shellfish harvesting (Q4) than participants in Sitka or Juneau. In terms of harvesting experience (Q7), again, more participants in Hoonah reported harvesting post‐program than participants in other communities, although levels of harvesting experience were also high post‐program among participants in Sitka, where the SEATOR lab is located. Post‐program, participants in Sitka were more likely to report behavioral beliefs that checking the SEATOR website would be rewarding (Q9) and normative beliefs that the people they harvest with would approve of them checking the SEATOR website (Q11). Post‐program, participants in Hoonah were least likely to report normative beliefs that the people they harvest with would approve of them checking the SEATOR website (Q11). Participants in Hoonah were also least likely to report control beliefs that participating in harvesting was up to them (Q5).

#### 3.1.4 Demographic Differences

Pre‐program, Alaska Native participants reported greater participation (Q7) and peer participation (Q4) in harvesting than non‐Native participants. However, Alaska Native participants also reported less confidence that they could check toxin levels before harvesting (Q13) and lower intentions to use the website to make harvesting decisions (Q15) than non‐Native participants pre‐program. We did not see statistically significant differences in pre‐post‐program changes in TPB beliefs and behavioral intentions between Alaska Native and non‐Native participants. However, we observed less variation between Alaska Native and non‐Native participants in the post‐program survey, which suggests that the program was successful in reaching all students. For example, pre‐program differences between Alaska Native and non‐Native participants related to confidence in checking the SEATOR website (Q13) and intentions to check the website (Q15) were less pronounced post‐program. Differences in perceptions of harvesting between Alaska Native and non‐Native participants also narrowed post‐program. Despite convergence in these questions, Alaska Natives continued to report more frequent peer participation in shellfish harvesting (Q4). We did not observe differences by gender.

#### 3.1.5 One Year Post‐Program Follow‐Up

Across the four respondents to the 1‐year post‐program survey, participation in shellfish harvesting and intentions to check the SEATOR website for toxin levels increased slightly, on average, compared to the post‐program survey. Past use of the SEATOR website and willingness to participate in harvesting remained constant.

### 3.2 Interview Results

#### 3.2.1 Behavioral Beliefs

Interviews reveal rationales behind shellfish harvesting and consumption and toxin exposure risk reduction beliefs and planned behaviors. In describing why they enjoyed shellfish harvesting, participants stressed the social aspects of harvesting with and for family members:

It [shellfish harvesting] gets me and my family closer.

(participant 13)

I know that my grandma enjoys it [shellfish harvesting] because she gets to hang out with all her friends while also getting food.

(participant 22)

In addition to spending time with family and friends and providing food for their families, participants commented that they appreciated the opportunity to connect with their culture and be outdoors and described food security and health benefits of harvesting. As one participant described the harvesting rationale of a family member:

I think he [family member] goes [harvesting] because I guess it saves money, and he always says it’s healthier and going out to get them is good exercise.

(participant 18)

Participants who did not harvest commented that they did not know how, did not have someone to take them, had experienced poor weather when harvesting previously, or were not interested. However, participants with little or no harvesting experience also reported that they felt that they would enjoy harvesting if they had opportunities to take part in and learn the process (e.g., participant 5). It is worth noting that participants rarely mentioned toxin exposure concerns as a reason not to harvest. In discussing why they consumed shellfish, participants mostly cited personal preferences while also emphasizing social and family motivations for harvesting. For example, one participant who harvested and ate shellfish recounted:

It [shellfish harvesting] is fun, and the clams taste really good when you do it the right way.

(participant 29)

Another participant who harvested but did not eat shellfish remarked:

I just don’t like them in general. I harvest them for my family.

(participant 19)

#### 3.2.2 Normative Beliefs

Family members' harvesting perceptions and practices had a strong influence on participants' perceptions and behaviors, both before and after the program. Although few participants had harvested prior to the program, those with harvesting experience and who reported enjoying harvesting had all previously harvested with family members. For participants who had harvested with family, the involvement of family members, often parents, made them feel safe about harvesting (e.g., participants 29, 34). Harvesting with family appeared to increase following the program, and, post‐program, participants reported greater awareness of family members' perceptions of shellfish harvesting, suggesting that participants may have discussed shellfish harvesting with family members during the program. Participants' experiences and learning in the program may also have influenced parents' shellfish harvesting beliefs and behaviors. Participants discussed sharing harvesting knowledge with family members and directing family members to the SEATOR website. Parents also reported to educational coordinators that, through their children's participation in the program, they had learned shellfish harvesting techniques and toxin exposure risk reduction strategies. According to one parent, their child's new interest in shellfish harvesting led them to take their children harvesting. Following processes their child had learned, they helped their children to record the time, date, location, and coordinates of their harvest and send clams to the SEATOR lab for toxin testing. Other participant comments reflect the bidirectional influence of normative beliefs, with parents' perceptions influencing children's perceptions and children's new knowledge from the program contributing to parents' shellfish harvesting and toxin awareness:

My parents said that it would be really cool to learn about the land, so we can eventually go and harvest clams. And it was cool that I was learning about the toxins, because they [my parents] didn’t know about that before.

(participant 25)

#### 3.2.3 Control Beliefs

Interviews emphasized the impact of program learning on risk reduction‐related knowledge, perceptions, and planned behaviors. Several participants stressed that their new understanding of poisoning risks made them more likely to use the risk reduction resources and strategies they had learned in the program:

I didn't even know about toxins before, so I feel like I’m more likely to check the website.

(participant 5)

[I am] more cautious, and I would definitely use the website if I were to go harvesting in the future.

(participant 15)

Participants also expressed confidence in their abilities to pursue risk reduction strategies. For example, illustrating familiarity with multiple risk reduction strategies, one participant described both checking toxin levels on the SEATOR website and sending samples to the SEATOR lab for toxin testing:

[You can] look up the right time of year on that website, look up the lethal exposures, which types of shellfish poisoning cause different symptoms, and you can go and get them [shellfish] tested at the Tribe.

(participant 8)

Participants' post‐program familiarity with SEATOR's risk reduction resources and belief in their ability to use these resources led participants to feel safe when consuming shellfish:

I feel that the process is pretty safe, but you got to check everything is safe to eat clams.

(participant 6)

Noting their intentions to check the SEATOR website for toxin levels before harvesting, participants shared that it was because of this strategy that they felt safe harvesting:

[The SEATOR website] will help me and my family not get sick.

(participant 19)

I feel safe as long as I look at the SEATOR website and see if it’s good to go.

(participant 5)

#### 3.2.4 Site and Demographic Differences

Interviews reveal possible explanations for the site and demographic differences observed in surveys. For example, related to the pre‐program difference in harvesting experiences between Alaska Native and non‐Native participants, some non‐Native participants discussed having moved to Alaska recently and lacking peer and family connections to harvesting as contributing to them not having harvested (e.g., participant 5). Alaska Native participants described social and familial aspects of harvesting and emphasized harvesting's cultural significance. Related to the pre‐program difference in harvesting experiences across sites, where participants in Hoonah were more likely to report harvesting experience than participants in Sitka and Juneau, participants in Hoonah especially emphasized that they harvested to provide food for family meals and community gatherings, commenting that they harvested “for food” (participant 17), “for subsistence, to eat” (participant 14), and “for family dinners” (participant 26).

## 4 Discussion

Overall, our results indicate more positive perceptions and increased behavioral intentions toward shellfish harvesting and checking the SEATOR website for toxin levels post‐program, although differences emerged across sites and by Alaska Native identity. Several key findings emerged: participants' understanding of the risk reduction strategy and confidence in their ability to check the SEATOR website increased and suggests that targeting control beliefs and offering an accessible risk reduction strategy is effective in shifting health and environmental behaviors. Participants also indicated that family involvement in harvesting shaped their perceptions, underscoring the role of normative beliefs and the importance of family engagement in children's environmental education. Taken together, findings emphasize that culturally tailored environmental education that targets multiple TPB beliefs and integrates risk perception and practical risk reduction strategies within local ecological and cultural contexts can influence children's health‐supporting planned behaviors. Several limitations might be considered when assessing findings, including that self‐reported data are subject to inaccuracies and bias (Brewer et al., 2007) and that differences in program implementation across communities may have affected participant responses, reflecting the challenges of standardizing interventions in diverse settings.

### 4.1 Environmental Health Knowledge

Results reveal post‐program increases in understanding of shellfish toxins and exposure risks, positive attitudes toward harvesting, intentions to use toxin risk reduction resources, and feelings of safety when harvesting. TPB research emphasizes the role of improving attitudes toward health‐supporting behaviors in contributing to behavior change (Ajzen, 1991; Fishbein & Ajzen, 2011). Our findings, where environmental health knowledge, attitudes, and planned behaviors all increased post‐program, similarly suggest the important role of new knowledge in shifting perceptions and behavioral intentions.

### 4.2 Behavioral Beliefs

Behavioral beliefs reflect beliefs about what actions are effective in making a positive difference. We observed the largest pre‐post‐program shifts in behavioral belief questions. Specifically, we observed greater agreement post‐program that checking the SEATOR website for toxin levels is rewarding and easy. Similar increases in perceptions that activities are safe and enjoyable have been observed in other children's environmental education contexts (Sommer & Kouba, 2015; Steg & Vlek, 2009). Interviews revealed that cultural and social factors may play a critical role in shaping behavioral beliefs. The cultural significance of clam harvesting was repeatedly raised as a major influence on desires to harvest and harvest safely, emphasizing the effectiveness of traditional knowledge in enhancing engagement and health outcomes with Indigenous participants (McOliver et al., 2015).

### 4.3 Normative Beliefs

Normative beliefs reflect beliefs about what is culturally and socially acceptable. Family harvesting experience emerged as a major influence on harvesting perceptions and behaviors, particularly pre‐program behaviors. In interviews, many Alaska Native participants discussed harvesting as a family activity that reinforced cultural traditions and strengthened community bonds. This description aligns with extensive research on the influence of social context and social norms on individual health, eating, and environmental behaviors (Bamberg & Möser, 2007; Conner & Armitage, 1998; De Leeuw et al., 2015; Steg & Vlek, 2009; Stok et al., 2016). Normative influences during the program seemed bidirectional, with parents' beliefs influencing participants' beliefs and participants' learning influencing parents' beliefs and behaviors. Thus, both participants and their families may have benefited from program lessons, which is consistent with previous research on family engagement in health education (Blue Bird Jernigan et al., 2012; Wexler et al., 2014).

### 4.4 Control Beliefs

Control beliefs reflect beliefs about what changes are within an individual's power to achieve. Participants reported an increase in knowledge and positive perceptions of risk reduction strategies and resources post‐program, with knowledge and agency related to toxin exposure risk reduction among the largest positive shifts. Positive perceptions of SEATOR resources especially increased, and participants reported that their greater familiarity with SEATOR resources and confidence in their ability to use these resources led them to feel safer when harvesting. Other research has noted similarly large shifts in control beliefs, as well as corresponding shifts in planned health behaviors (Ferrer & Klein, 2015; Slovic et al., 2013), underscoring the role of reliable information and access to this information in influencing behavioral intentions (Turra et al., 2016).

### 4.5 Behavioral Intentions and Behavioral Outcomes

Post‐program responses indicate overall greater willingness to harvest and check the SEATOR website for toxin levels, reflecting the education program's success in encouraging safe shellfish harvesting. Shifts in multiple types of TPB beliefs corresponded with shifts in both harvesting and risk reduction behavioral intentions. These accompanying changes in behavioral intentions suggest that targeting multiple TPB beliefs may be an effective approach to influence planned behaviors.

### 4.6 Site and Demographic Differences

Among sites, responses differed most between participants in Hoonah and participants in Juneau and Sitka. Pre‐program, Hoonah participants were more likely to hold positive perceptions of harvesting and to have harvested. Hoonah is the smallest and most remote community in this study and, compared with larger and more connected Sitka and Juneau, imported foods may be more expensive and less accessible. Hoonah also has a larger proportion of Alaska Native residents (33% compared to 9% in both Sitka and Juneau) (U.S. Census Bureau, 2022), and Alaska Natives are more likely to harvest and consume shellfish than non‐Natives. Responses of Hoonah participants also differed from Juneau and Sitka participants in the post‐program survey. Compared to Juneau and Sitka participants, Hoonah participants reported less agreement that harvesting decisions were up to them and that the people they harvested with would approve of them checking the SEATOR website. These differences may reflect harvesting as a more common practice in Hoonah as long‐time harvesters may be slower to adopt new harvesting habits such as checking the SEATOR website. These differences may also reflect harvesting as a more common family activity in Hoonah as participants may feel more obliged to harvest, and harvest in a certain way, with family members.

We also observed differences between Sitka participants and Juneau and Hoonah participants. Post‐program, Sitka participants reported especially positive perceptions of checking the SEATOR website. The SEATOR lab is in Sitka, and this proximity and Sitka participants' hands‐on experience with the lab during the program may have contributed to particularly positive perceptions of risk reduction strategies post‐program compared to participants in Juneau and Hoonah. This difference highlights the value of local resources in health interventions, also noted in other studies (Brewer et al., 2007; Shi & Kim, 2020). Although localized toxin testing is not feasible in this context, environmental managers across Southeast Alaska have called for small and remote communities to have greater access to resources, services, and programming, including environmental education opportunities such as the program discussed in this study (Roland, Kohlhoff, Lanphier, Hoysala, et al., 2024).

Several differences emerged between Alaska Native and non‐Native participants. Alaska Native participants were more likely to have harvested pre‐program and to report positive peer perceptions of harvesting, both pre‐ and post‐program. These differences likely reflect shellfish harvesting as a traditional practice among Alaska Natives, as well as non‐Native participants' limited exposure to harvesting, which may in turn reflect ongoing Alaska Native—White segregation (Cole, 2020). Related to toxin exposure risk reduction, Alaska Native participants reported less self‐efficacy and interest in checking the SEATOR website for toxin levels compared to non‐Native participants. As with differences observed across sites, these differences could indicate more frequent harvesting with family members and deference to relatives' harvesting habits.

Site and demographic differences were noticeable both pre‐ and post‐program, but we also observed post‐program convergence. Participant convergence may reflect the success of the program in shifting students' perceptions and planned behaviors across sites and demographic groups, which is supported by related research in the region that demonstrates the effectiveness of community‐based interventions in contributing to behavioral change across groups (Harley et al., 2020).

## 5 Conclusions

This study advances our understanding of how TPB beliefs, and particularly control beliefs, can be targeted to influence healthy behaviors. The success of the program in enhancing participants' knowledge of the risk reduction strategy and confidence in carrying out the strategy suggests that providing accessible risk reduction strategies is effective in promoting safe behaviors. Further, this study suggests that integrating risk perception into the TPB framework by emphasizing risks associated with exposures while promoting self‐efficacy in risk reduction can align perceived risks with behavioral intentions and support safe practices. Future research on strategies to modify health behaviors might consider addressing different types of risk perceptions, as tailoring risk perceptions and efficacy beliefs may increase impacts on behavior change (Brewer et al., 2007; Ferrer & Klein, 2015).

Our study emphasizes that environmental education programs designed to fit cultural norms and address local environmental and health concerns can positively influence behavioral intentions, suggesting the broader applicability of culturally informed literacy programs in promoting public health and environmental stewardship. Indigenous communities' traditions, practices, and perceptions influence how educational interventions are implemented and received, and tailoring programs to these contexts can enhance relevance, engagement, and outcomes (Manson et al., 2004). For example, incorporating traditional knowledge and cultural practices into curricula can make content more relatable and impactful (Armstrong et al., 2007), as it did in our study. A context‐directed approach not only respects and preserves cultural heritages but also leverages cultures to promote health and safety.

By centering sociocultural and environmental contexts and a conceptual model for behavioral change, the education program in this study may serve as a template for EHL programming that is both theoretically grounded and pragmatically effective in diverse settings (Finn & O’Fallon, 2017). The education program offers an example of how the mixture of knowledge systems, including traditional knowledge, local knowledge, and western science, may positively impact cultural integrity, self‐determination, and revitalization or continuation of customary cultural activities. Further, the hands‐on and experiential activities of the program may contribute to examples of transformative learning that introduce new information and resources, reframe perceived problems, promote confidence and discriminating decision making, and, ultimately, alter habits (Friedman, 2022). To facilitate the use and adaptation of the program's curriculum materials in environmental and cultural education in other communities and contexts, materials have been shared with communities across Southeast Alaska and on the National Institute of Environmental Health Sciences Partnerships for Environmental Public Health Resource Center (The National Institute of Environmental Health Sciences, n.d.).

Future studies may investigate roles of family and peer influences in contributing to behavior changes across diverse populations. Our findings emphasize the impact of family and social norms on attitudes and planned behaviors, including bidirectional influences and possible restrictive influences of family members' harvesting habits on health‐supporting behavior modifications. Engaging families and communities in the educational process may reinforce positive behaviors among both children and their families. Future studies may also explore knowledge retention and long‐term impacts of changes in TPB beliefs on risk reduction behaviors. As sustained engagement and reinforcement over time are critical for lasting behavioral change (Minkler & Wallerstein, 2012; Wallerstein & Duran, 2010), programs might consider incorporating follow‐up activities and ongoing support to help participants maintain the knowledge and behaviors they have learned. This could include community events and educational activities and periodic refresher programs similar to STA's elementary, middle, and high school environmental education programs.

## Conflict of Interest

The authors declare no conflicts of interest relevant to this study.

## Data Availability

Data are sensitive and confidential, so data were not deposited and are not available. Specifically, data include sensitive information about children's perceptions and planned behaviors related to a culturally important practice, and consent did not include permission for parties beyond the project team to use data for research or other purposes.

## Appendix A Consolidated Criteria for Strengthening Reporting of Health Research Involving Indigenous Peoples: The CONSIDER Statement (Huria et al., 2019)

**Table:**

Governance	
1. Describe partnership agreements between the research institution and Indigenous‐governing organization for the research, (e.g., Informal agreements through to MOU (Memorandum of Understanding) or MOA (Memorandum of Agreement))	This study is a collaboration between researchers at the University of Alabama at Birmingham (UAB), the University of California, San Francisco (UCSF), and the Sitka Tribe of Alaska (STA). The community‐academic partnership was initiated in 2016 when academic partners were invited to support the efforts of a tribally led environmental monitoring program (SEATOR) and a Tribe‐educational system partnership between the Sitka Tribe of Alaska and the Sitka School District. Staff working with the Central Council of the Tlingit and Haida Indian Tribes of Alaska and the Hoonah Indian Association collaborated in this education program and research, after expressing interest in expanding the program to their communities
2. Describe accountability and review mechanisms within the partnership agreement that addresses harm minimization	In the funding supporting this research, tribal organization representatives are included only as other formal co‐Investigators, indicating shared leadership with the grant PI, Dr. Gribble. Tribal organization representatives had the final say in all use of data and all research products
3. Specify how the research partnership agreement includes protection of Indigenous intellectual property and knowledge arising from the research, including financial and intellectual benefits generated (e.g., development of traditional medicines for commercial purposes or supporting the Indigenous community to develop commercialization proposals generated from the research)	The education materials designed and used as part of this study are the property of STA and were shared with other Southeast Alaska communities and on the National Institute of Environmental Health Sciences Partnerships for Environmental Public Health web resource database
Prioritization	
4. Explain how the research aims emerged from priorities identified by either Indigenous stakeholders, governing bodies, funders, non‐government organization(s), stakeholders, consumers, and empirical evidence	The goals of this project were developed during multiple site visits by Dr. Gribble to meet with STA staff and identify community and tribal needs and opportunities
Relationships (Indigenous stakeholders/participants and Research team)	
5. Specify measures that adhere and honor Indigenous ethical guidelines, processes, and approvals for all relevant Indigenous stakeholders, recognizing that multiple Indigenous partners may be involved, for example, Indigenous ethics committee approval, regional/national ethics approval processes	The UAB IRB was responsible for this research (IRB protocol number 300006786). All members of tribal organizations involved in the education program were included in the IRB, indicating involvement in research activities and access to data. Research processes (e.g., recruitment and consent) were developed with input from tribal representatives to ensure that they were appropriate to the participants and communities
6. Report how Indigenous stakeholders were involved in the research processes (i.e., research design, funding, implementation, analysis, and dissemination/recruitment)	STA staff designed the education program and the specific educational activities in the program. Coauthors with STA, the Central Council of the Tlingit and Haida Indian Tribes of Alaska, and the Hoonah Indian Association recruited and consented participants, implemented the education program, and supported data collection for the related research activities. With academic partners, STA staff conducted analysis of interview data and drafted interview results
7. Describe the expertise of the research team in Indigenous health and research	Hugh Roland is an Assistant Professor at UAB. He is an environmental sociologist with training in qualitative and statistical research methods and experience in climate and health research with Indigenous communities
	Jacob Kohlhoff was the Environmental Education Coordinator with STA when the education program described in this manuscript was designed and implemented. He has experience implementing education programs and was trained in interview and survey research techniques by Hugh Roland
	Travis Moore is a Postdoctoral Fellow at Tufts University. He is a human ecologist and systems scientist with expertise in the Theory of Planned Behavior and has experience in community‐based health research with Indigenous communities
	Kari Lanphier is an Environmental Program Manager with SEATOR and has expertise in harmful algal blooms, community program management, and shellfish toxin risk communication
	Lindsey Pierce is the Environmental Coordinator with the Central Council of the Tlingit and Haida Indian Tribes of Alaska's Environmental Division
	Julian Narvaez is the Environmental Education Coordinator with the Hoonah Indian Association
	Aissa Yazzie is faculty at Northwest Indian College. She has expertise in Indigenous methodologies and extensive experience with Indigenous health research
	Christopher Whitehead is an Environmental Program Manager with STA. He has expertise in harmful algal blooms and community program development.
	Jeff Feldpausch is the Resource Protection Director with STA
	Matthew Gribble is an Associate Professor at UCSF. He is an environmental epidemiologist with experience in community‐based participatory research and statistical methods
Methodologies	
8. Describe the methodological approach of the research including a rationale of methods used and implication for Indigenous stakeholders, for example, privacy and confidentiality (individual and collective)	The interview and survey methods used in this study were selected with the input of tribal organization representatives to capture both quantitative changes in beliefs and planned behaviors (surveys) and explanations for these changes (interviews). Confidentiality was protected by de‐identifying data and using a protected key to link study IDs to participant names
9. Describe how the research methodology incorporated consideration of the physical, social, economic, and cultural environment of the participants and prospective participants. (e.g., impacts of colonization, racism, and social justice), as well as Indigenous worldviews	The educational intervention was designed to emphasize the importance of Tlingit culture and the Southeast Alaska environment and support the use of an adaptation strategy developed and run by regional tribal organizations. The survey instrument and interview protocol used in the related research included questions about social and cultural influences on beliefs and planned behaviors
Participation	
10. Specify how individual and collective consent was sought to conduct future analysis on collected samples and data (e.g., additional secondary analyses; third‐parties accessing samples (genetic, tissue, blood) for further analyses)	Written consent was received from participants' parents prior to the conduct of research activities and from participants before the start of each research activity. Consent included the use of data for research to be conducted by the study team, which includes members of STA, the Central Council of the Tlingit and Haida Indian Tribes of Alaska, and the Hoonah Indian Association, and members of the study team may use study data in further analyses. Consent did not include permission for other parties to use data for research or other purposes as data includes sensitive information about children's perceptions and planned behaviors related to a culturally important practice
11. Described how the resource demands (current and future) placed on Indigenous participants and communities involved in the research were identified and agreed upon including any resourcing for participation, knowledge, and expertise	To reduce participant research burden and time taken from education program activities, the survey instrument and interview protocol were designed to enable relatively quick data collection, and the frequency of data collection was limited to the beginning and end of the program. Research participants received up to $100 for participating in the education program, $10 for each of the 10 sessions attended. Grant funds covered the cost of materials needed for the program. Tribal organizations received the majority of funds from the grant supporting this research. Grant funds were used to support the salaries of existing staff as well as support new staff to carry out the required activities
12. Specify how biological tissue and other samples including data were stored, explaining the processes of removal from traditional lands, if done, and of disposal	Collection and storage of original data and consent documentation was managed by members of STA, the Central Council of the Tlingit and Haida Indian Tribes of Alaska, and the Hoonah Indian Association. Original data were stored in the office of the Sitka Tribe of Alaska Resource Protection Department. De‐identified electronic data were stored on a password protected UAB Box drive. Data were only shared per community‐developed protocols
Capacity	
13. Explain how the research supported the development and maintenance of Indigenous research capacity (e.g., specific funding of Indigenous researchers)	Over the course of the project, coauthors received training in research methods and gained experience with survey and interview research. Grant funds supported two Indigenous coauthors, Kari Lanphier and Aissa Yazzie. Grant funds also supported the shellfish toxin testing program run by SEATOR, which enables the adaptation strategy targeted in the education program in this study
14. Discuss how the research team undertook professional development opportunities to develop the capacity to partner with Indigenous stakeholders?	Members of the research team attended the annual Southeast Environmental Conference organized by STA and the Central Council of the Tlingit and Haida Indian Tribes of Alaska where they learned about the work and needs of tribes across Southeast Alaska and met tribal organization representatives, sharing information about the education program and leading to the program's expansion to two new communities
Analysis and interpretation	
15. Specify how the research analysis and reporting supported critical inquiry and a strength‐based approach that was inclusive of Indigenous values	The education program was designed to support cultural continuity related to shellfish harvesting and included learning an adaptation strategy developed and led by tribal organizations in the region. Analysis and reporting were sensitive to this goal and to Alaska Native leadership in climate and health adaptation. Analysis involved members of the author team with academic and tribal affiliations and included regular meetings to review analysis and findings
Dissemination	
16. Describe the dissemination of the research findings to relevant Indigenous governing bodies and peoples	Members of the author team with tribal affiliations will share findings with their organizations and communities as they feel is appropriate
17. Discuss the process for knowledge translation and implementation to support Indigenous advancement (e.g., research capacity, policy, and investment)	In sharing study findings with their organizations, members of the author team with tribal affiliations may note how findings can be applied to and inform other programs. For example, this research may inform STA's other environmental education programs, including elementary and high school programs. These programs may be refined based on study findings, for example, to include the targeting of a specific adaptation strategy and to further encourage family and community participation in learning

## Appendix B Survey Responses by Site, Alaska Native Identity, and Gender Identity. Likert‐Scale Responses Ranged From Disagree (1) to Agree (5)

**Table:**

	Site						Alaska native identity				Gender identity						Total	
	Pre			Post			Pre		Post		Pre			Post			Pre	Post
	Hoonah *N* = 20	Juneau *N* = 19	Sitka *N* = 11	Hoonah *N* = 20	Juneau *N* = 19	Sitka *N* = 11	AN *N* = 27	Non‐AN *N* = 20	AN *N* = 27	Non‐AN *N* = 20	F *N* = 18	M *N* = 21	NB *N* = 6	F *N* = 18	M *N* = 21	NB *N* = 6	*N* = 50	*N* = 50
Q1 (Behavioral beliefs): Subsistence clam harvesting is enjoyable																		
*N* missing	0	8	3	0	2	0	3	5	0	2	2	4	2	0	2	0	11	2
Mean	3.6	3.5	3.8	4.1	4.1	4.4	3.6	3.6	4.2	4.2	3.5	3.8	3.5	4.1	4.2	4.2	3.6	4.2
(SD)	(0.8)	(0.5)	(1.5)	(0.8)	(0.6)	(1.0)	(0.8)	(1.1)	(0.7)	(0.9)	(1.0)	(0.8)	(0.6)	(0.9)	(0.8)	(0.8)	(0.9)	(0.8)
Range	2.5–5	3–4	1–5	2.5–5	3–5	2–5	3–5	1–5	3–5	2–5	1–5	2.5–5	3–4	2–5	2.5–5	3–5	1–5	2–5
Q2 (Behavioral beliefs): Subsistence clam harvesting is rewarding																		
*N* missing	0	8	3	0	3	0	3	5	1	2	2	4	2	1	2	0	11	3
Mean	4.6	3.7	3.5	4.3	4.2	4.0	4.4	3.7	4.2	4.2	4.2	3.9	4.8	4.5	3.9	4.3	4.1	4.2
(SD)	(0.8)	(0.9)	(1.3)	(0.8)	(1.1)	(1.0)	(0.9)	(1.2)	(1.0)	4.2	(1.2)	(1.0)	(0.5)	(0.7)	(1.2)	(0.8)	(1.0)	(1.0)
Range	3–5	2–5	1–5	3–5	1–5	3–5	3–5	1–5	1–5	3–5	1–5	2–5	4–5	3–5	1–5	3–5	1–5	1–5
Q3 (Normative beliefs): Most of the people who are important to me would approve of me participating in subsistence clam harvesting																		
*N* missing	0	7	0	0	2	0	1	3	0	2	2	1	1	0	2	0	7	2
Mean	4.4	3.9	4.5	4.4	4.4	4.5	4.3	4.3	4.4	4.4	4.4	4.2	4.4	4.4	4.4	4.5	4.3	4.4
(SD)	(1.1)	(0.9)	(0.8)	(0.8)	(0.6)	(0.5)	(1.1)	(0.9)	(0.7)	(0.7)	(1.1)	(0.9)	(0.9)	(0.6)	(0.7)	(0.8)	(1.0)	(0.7)
Range	1–5	3–5	3–5	2.5–5	3–5	4–5	1–5	2.5–5	3–5	2.5–5	1–5	2.5–5	3–5	3–5	2.5–5	3–5	1–5	2–5
Q4 (Normative beliefs): Most of my peers (classmates, friends, etc.) participate in subsistence clam harvesting																		
*N* missing	0	7	0	0	2	0	1	3	0	2	2	1	1	0	2	0	7	2
Mean	3.6	2.6	2.8	3.7	2.6	3.0	3.4	2.7	3.6	2.7	3.6	2.9	3.0	3.1	3.4	3.5	3.1	3.1
(SD)	(1.1)	(1.2)	(1.2)	(1.0)	(1.3)	(1.2)	(1.2)	(1.1)	(1.1)	(1.3)	(1.2)	(1.3)	(0.7)	(1.1)	(1.4)	(0.8)	(1.2)	(1.2)
Range	1–5	1–4	1–5	2–5	1–5	1–5	1–5	1–4	1–5	1–5	1–5	1–5	2–4	1–5	1–5	3–5	1–5	1–5
Q5 (Control beliefs): Whether or not I participate in subsistence clam harvesting is up to me																		
*N* missing	0	7	0	1	2	0	1	3	1	2	2	1	1	0	3	0	7	3
Mean	3.9	4.4	4.5	4.0	4.8	4.6	4.0	4.4	4.3	4.6	4.3	4.1	4.2	4.5	4.3	4.5	4.2	4.4
(SD)	(1.5)	(0.9)	(0.7)	(1.1)	(0.4)	(0.5)	(1.2)	(1.1)	(0.8)	(1.0)	(1.4)	(1.2)	(0.8)	(0.7)	(1.1)	(0.8)	(1.2)	(0.9)
Range	1–5	3–5	3–5	1–5	4–5	4–5	1–5	1–5	3–5	1–5	1–5	1–5	3–5	3–5	1–5	3–5	1–5	1–5
Q6 (Behavioral intention): I intend to participate in subsistence clam harvesting this year																		
*N* missing	0	7	0	0	3	0	1	3	0	3	2	1	1	0	2	1	7	3
Mean	3.6	4.1	4.5	3.6)	3.1	3.4	3.9	4.1	3.7	3.1	3.8	4.2	4.0	3.2	3.7	3.8	4.0	3.4
(SD)	(1.3)	(1.3)	(1.3)	(1.3)	(1.5)	(1.5)	(1.1)	(1.6)	(1.2)	(1.5)	(1.4)	(1.3)	(1.0)	(1.4)	(1.4)	(1.1)	(1.3)	(1.4)
Range	1–5	1–5	1–5	1–5	1–5	1–5	1–5	1–5	1–5	1–5	1–5	1–5	3–5	1–5	1–5	3–5	1–5	1–5
Q7 (Behavior): I have participated in subsistence clam harvesting																		
*N* missing	0	8	0	0	3	0	1	4	0	3	2	2	1	0	2	1	8	3
Mean	3.5	1.4	1.9	3.6	1.3	3.5	3.2	1.6	3.3	2.4	2.8	2.2	3.4	3.2	2.6	4.2	2.5	2.8
(SD)	(1.9)	(0.8)	(1.3)	(1.7)	(0.9)	(1.4)	(1.9)	(1.2)	(1.7)	(1.7)	(1.9)	(1.8)	(1.8)	(1.9)	(1.7)	(0.8)	(1.8)	(1.8)
Range	1–5	1–3	1–5	1–5	1–4	1–5	1–5	1–5	1–5	1–5	1–5	1–5	1–5	1–5	1–5	3–5	1–5	1–5
Q8 (Behavioral intention): Since learning about clam harvest safety, I feel more willing to participate in subsistence clam harvesting																		
*N* missing				0	3	0			0	3				0	2	1		3
Mean				4.2	4.6	4.4			4.4	4.4				4.6	4.1	4.6		4.4
(SD)				(1.1)	(0.6)	(0.8)			(0.9)	(0.9)				(0.7)	(1.0)	(0.9)		(0.9)
Range				2–5	3–5	3–5			2–5	2–5				3–5	2–5	3–5		2–5
Q9 (Behavioral beliefs): Checking the SEATOR website before clam harvesting would be rewarding																		
*N* missing	0	12	1	0	2	0	4	6	0	2	4	4	1	0	2	0	13	2
Mean	3.4	3.3	3.9	4.0	4.4	4.7	3.6	3.5	4.3	4.4	3.5	3.5	3.6	4.4	4.2	4.5	3.5	4.3
(SD)	(1.5)	(0.5)	(1.3)	(0.9)	(0.8)	(0.5)	(1.4)	(1.0)	(0.8)	(0.7)	(1.5)	(1.1)	(1.7)	(0.8)	(0.8)	(0.8)	(1.3)	(0.8)
Range	1–5	3–4	2–5	3–5	3–5	4–5	1–5	2–5	3–5	3–5	1–5	1–5	1–5	3–5	3–5	3–5	1–5	3–5
Q10 (Control beliefs): Checking the SEATOR website before clam harvesting would be easy																		
*N* missing	0	12	1	0	2	0	4	6	0	2	4	4	1	0	2	0	13	2
Mean	3.9	3.4	3.9	4.0	4.2	4.5	3.7	3.9	4.1	4.3	4.1	3.6	3.6	4.0	4.3	4.3	3.8	4.2
(SD)	(1.1)	(1.3)	(1.2)	(0.9)	(1.0)	(0.7)	(1.1)	(1.3)	(0.8)	(1.0)	(1.1)	(1.3)	(0.5)	(0.9)	(0.9)	(0.8)	(1.2)	(0.9)
Range	1–5	2–5	2–5	3–5	2–5	3–5	1–5	2–5	3–5	2–5	2–5	1–5	3–4	2–5	3–5	3–5	1–5	2–5
Q11 (Normative beliefs): The people who I go clam harvesting with would approve of me checking the SEATOR website before clam harvesting																		
*N* missing	0	12	3	0	2	0	6	6	0	2	4	5	2	0	2	0	15	2
Mean	3.5	4.1	4.5	3.9	4.5	4.8	3.7	4.1	4.2	4.6	3.7	3.9	3.8	4.5	4.3	3.8	3.9	4.3
(SD)	(1.3)	(0.9)	(1.1)	(1.0)	(0.7)	(0.4)	(1.2)	(1.3)	(0.9)	(0.7)	(1.2)	(1.2)	(1.9)	(0.8)	(0.9)	(1.0)	(1.2)	(0.9)
Range	1–5	3–5	2–5	2.5–5	3–5	4–5	1–5	1–5	2.5–5	3–5	1–5	1–5	1–5	3–5	2.5–5	3–5	1–5	2.5–5
Q12 (Normative beliefs): Most people like me (classmates, friends, etc.) will check the SEATOR website before clam harvesting																		
*N* missing	0	12	1	0	2	0	4	6	0	2	4	4	1	0	2	0	13	2
Mean	2.9	3.3	3.8	3.3	3.4	4.0	3.1	3.4	3.3	3.9	2.9	3.4	3.2	3.9	3.1	3.7	3.2	3.5
(SD)	(1.3)	(1.3)	(1.1)	(1.1)	(1.5)	(1.3)	(1.2)	(1.4)	(1.2)	(1.3)	(1.2)	(1.2)	(1.5)	(1.1)	(1.5)	(1.0)	(1.3)	(1.3)
Range	1–5	2–5	2–5	1–5	1–5	1–5	1–5	1–5	1–5	1–5	1–5	1–5	1–5	1–5	1–5	3–5	1–5	1–5
Q13 (Control beliefs): I am confident that I can check the SEATOR website before clam harvesting																		
*N* missing	0	12	0	0	2	0	4	5	0	2	4	3	1	0	2	0	12	2
Mean	3.3	3.6	4.2	3.5	4.1	4.1	3.3	4.1	3.8	4.1	3.3	3.8	3.6	3.9	3.6	4.3	3.6	3.9
(SD)	(1.2)	(1.5)	(0.8)	(1.2)	(1.3)	(1.2)	(1.1)	(1.2)	(1.2)	(1.3)	(1.4)	(1.1)	(1.1)	(1.3)	(1.3)	(0.8)	(1.2)	(1.3)
Range	1–5	1–5	3–5	1–5	1–5	2–5	1–5	1–5	1–5	1–5	1–5	1–5	2–5	1–5	1–5	3–5	1–5	1–5
Q14 (Control beliefs): Checking the SEATOR website to decide if I should go clam harvesting is up to me																		
*N* missing	0	12	0	0	2	0	4	5	0	2	4	3	1	0	2	0	12	2
Mean	3.9	4.1	4.3	4.3	4.5	4.4	4.1	3.9	4.5	4.2	4.2	3.8	4.0	4.4	4.4	4.5	4.0	4.4
(SD)	(1.3)	(1.2)	(0.9)	(1.1)	(0.7)	(1.3)	(1.1)	(1.3)	(0.8)	(1.4)	(1.1)	(1.4)	(0.7)	(0.8)	(1.3)	(0.8)	(1.2)	(1.0)
Range	1–5	2–5	2–5	1–5	3–5	1–5	1–5	1–5	3–5	1–5	2–5	1–5	3–5	3–5	1–5	3–5	1–5	1–5
Q15 (Behavioral intention): I intend to check the SEATOR website to help decide if I should go clam harvesting																		
*N* missing	0	12	0	0	3	0	2	7	0	3	3	5	1	0	2	1	12	3
Mean	3.5	4.0	4.3	3.5	4.0	4.3	3.4	4.5	3.9	4.0	3.9	3.9	3.6	4.2	3.7	3.8	3.8	3.9
(SD)	(1.3)	(1.5)	(0.8)	(0.8)	(1.2)	(1.2)	(1.3)	(0.7)	(0.8)	(1.2)	(1.2)	(1.1)	(1.5)	(0.8)	(1.2)	(0.8)	(1.2)	(1.0)
Range	1–5	1–5	3–5	2–5	1–5	2–5	1–5	3–5	2–5	2–5	2–5	1–5	1–5	3–5	2–5	3–5	1–5	1–5
Q16 (Behavioral intention): Since I have found out about the resources available on the SEATOR website, I have used them when deciding whether to clam harvest																		
*N* missing				0	3	0			0	3				0	2	1		3
Mean				3.2	3.1	2.9			3.1	3.1				3.5	2.7	3.8		3.1
(SD)				(1.4)	(1.6)	(1.8)			(1.5)	(1.5)				(1.5)	(1.5)	(1.8)		(1.5)
Range				1–5	1–5	1–5			1–5	1–5				1–5	1–5	1–5		1–5

## Appendix C Differences in Theory of Planned Behavior Beliefs and Behavioral Intentions Between Pre‐ and Post‐Program Surveys From Generalized Estimating Equation Linear Regressions Accounting for Clustering of Longitudinal Observations Within Person, Adjusted Associations and 95% Confidence Intervals: Full Results Table

**Table:**

Overall association (95% CI)	Estimated associations by site (95% CI)		Estimated association by Alaska native identity (95% CI)		Estimated association by gender identity (95% CI)	
*p*‐value (*X* ^2^ test)	*p*‐value for interaction (*F* test)		*p*‐value for interaction (*F* test)		*p*‐value for interaction (*F* test)	
Q1 (Behavioral beliefs): Subsistence clam harvesting is enjoyable						
0.51 (0.16, 0.86) *p* = 0.01	Hoonah Juneau Sitka	0.53 (0.05, 1.00) 0.46 (−0.25, 1.17) 0.53 (−0.17, 1.24) *p* = 0.99	Alaska Native Non‐Native	0.45 (0.01, 0.89) 0.58 (0.02, 1.15) *p* = 0.72	Boy Girl Nonbinary	0.41 (−0.12, 0.94) 0.60 (0.06, 1.14) 0.56 (−0.41, 1.54) *p* = 0.88
Q2 (Behavioral beliefs): Subsistence clam harvesting is rewarding						
0.09 (−0.34, 0.52) *p* = 0.67	Hoonah Juneau Sitka	−0.25 (−0.82, 0.32) 0.26 (−0.56, 1.08) 0.50 (−0.34, 1.34) *p* = 0.33	Alaska Native Non‐Native	−0.14 (−0.67, 0.40) 0.40 (−0.27, 1.08) *p* = 0.22	Boy Girl Nonbinary	0.04 (−0.59, 0.67) 0.24 (−0.42, 0.89) −0.15 (−1.36, 1.05) *p* = 0.83
Q3 (Normative beliefs): Most of the people who are important to me would approve of me participating in subsistence clam harvesting						
0.13 (−0.15, 0.41) *p* = 0.35	Hoonah Juneau Sitka	0.05 (−0.34, 0.44) 0.28 (−0.28, 0.85) 0.09 (−0.43, 0.61) *p* = 0.77	Alaska Native Non‐Native	0.18 (−0.17, 0.53) 0.06 (−0.39, 0.52) *p* = 0.67	Boy Girl Nonbinary	0.25 (−0.16, 0.66) 0.01 (−0.43, 0.45) 0.09 (−0.69, 0.87) *p* = 0.74
Q4 (Normative beliefs): Most of my peers (classmates, friends, etc.) participate in subsistence clam harvesting						
0.09 (−0.43, 0.61) *p* = 0.73	Hoonah Juneau Sitka	0.05 (−0.70, 0.80) 0.08 (−0.94, 1.10) 0.18 (−0.83, 1.19) *p* = 0.98	Alaska Native Non‐Native	0.22 (−0.45, 0.88) −0.08 (−0.92, 0.76) *p* = 0.59	Boy Girl Nonbinary	0.45 (−0.30, 1.19) −0.44 (−1.24, 0.36) 0.44 (−0.96, 1.85) *p* = 0.25
Q5 (Control beliefs): Whether or not I participate in subsistence clam harvesting is up to me						
0.20 (−0.13, 0.52) *p* = 0.23	Hoonah Juneau Sitka	0.19 (−0.25, 0.64) 0.29 (−0.40, 0.99) 0.09 (−0.49, 0.67) *p* = 0.91	Alaska Native Non‐Native	0.26 (−0.15, 0.66) 0.12 (−0.42, 0.66) *p* = 0.70	Boy Girl Nonbinary	0.20 (−0.28, 0.69) 0.17 (−0.33, 0.67) 0.26 (−0.63, 1.16) *p* = 0.98
Q6 (Behavioral intention): I intend to participate in subsistence clam harvesting this year						
−0.42 (−0.96, 0.11) *p* = 0.12	Hoonah Juneau Sitka	0.05 (−0.67, 0.77) −0.58 (−1.64, 0.48) −1.09 (−2.06, −0.12) *p* = 0.22	Alaska Native Non‐Native	−0.17 (−0.84, 0.49) −0.77 (−1.63, 0.10) *p* = 0.29	Boy Girl Nonbinary	−0.38 (−1.15, 0.39) −0.56 (−1.39, 0.27) −0.18 (−1.75, 1.39) *p* = 0.90
Q7 (Behavior): I have participated in subsistence clam harvesting						
0.41 (−0.23, 1.05) *p* = 0.21	Hoonah Juneau Sitka	0.10 (−0.68, 0.88) −0.11 (−1.49, 1.27) 1.64 (0.59, 2.68) *p* = 0.08	Alaska Native Non‐Native	0.14 (−0.60, 0.89) 0.77 (−0.31, 1.86) *p* = 0.34	Boy Girl Nonbinary	0.29 (−0.62, 1.20) 0.43 (−0.52, 1.39) 0.77 (−1.17, 2.71) *p* = 0.89
Q9 (Behavioral beliefs): Checking the SEATOR website before clam harvesting would be rewarding						
0.71 (0.27, 1.15) *p* = 0.00	Hoonah Juneau Sitka	0.58 (−0.02, 1.17) 0.76 (−0.15, 1.68) 0.89 (0.07, 1.71) *p* = 0.84	Alaska Native Non‐Native	0.65 (0.10, 1.19) 0.80 (0.07, 1.53) *p* = 0.74	Boy Girl Nonbinary	0.67 (0.01, 1.34) 0.74 (0.05, 1.43) 0.77 (−0.42, 1.97) *p* = 0.98
Q10 (Control beliefs): Checking the SEATOR website before clam harvesting would be easy						
0.37 (0.00, 0.73) *p* = 0.05	Hoonah Juneau Sitka	0.15 (−0.32, 0.62) 0.54 (−0.22, 1.29) 0.55 (−0.12, 1.22) *p* = 0.57	Alaska Native Non‐Native	0.40 (−0.05, 0.84) 0.33 (−0.29, 0.94) *p* = 0.86	Boy Girl Nonbinary	0.63 (0.11, 1.14) −0.03 (−0.60, 0.53) 0.67 (−0.25, 1.58) *p* = 0.19
Q11 (Normative beliefs): The people who I go clam harvesting with would approve of me checking the SEATOR website before clam harvesting						
0.37 (−0.07, 0.82) *p* = 0.10	Hoonah Juneau Sitka	0.43 (−0.17, 1.02) 0.30 (−0.60, 1.19) 0.38 (−0.46, 1.23) *p* = 0.97	Alaska Native Non‐Native	0.34 (−0.21, 0.90) 0.42 (−0.29, 1.12) *p* = 0.88	Boy Girl Nonbinary	0.30 (−0.34, 0.94) 0.64 (−0.03, 1.30) −0.14 (−1.35, 1.07) *p* = 0.51
Q12 (Normative beliefs): Most people like me (classmates, friends, etc.) will check the SEATOR website before clam harvesting						
0.30 (−0.30, 0.91) *p* = 0.32	Hoonah Juneau Sitka	0.43 (−0.40, 1.25) 0.16 (−1.06, 1.38) 0.27 (−0.88, 1.42) *p* = 0.93	Alaska Native Non‐Native	0.11 (−0.64, 0.86) 0.56 (−0.40, 1.53) *p* = 0.47	Boy Girl Nonbinary	−0.21 (−1.06, 0.65) 0.85 (−0.06, 1.77) 0.45 (−1.12, 2.02) *p* = 0.24
Q13 (Control beliefs): I am confident that I can check the SEATOR website before clam harvesting						
0.22 (−0.25, 0.70) *p* = 0.36	Hoonah Juneau Sitka	0.20 (−0.45, 0.85) 0.51 (−0.48, 1.49) −0.09 (−0.96, 0.78) *p* = 0.66	Alaska Native Non‐Native	0.35 (−0.25, 0.94) 0.06 (−0.71, 0.83) *p* = 0.56	Boy Girl Nonbinary	−0.16 (−0.85, 0.52) 0.50 (−0.21, 1.22) 0.74 (−0.54, 2.03) *p* = 0.30
Q14 (Control beliefs): Checking the SEATOR website to decide if I should go clam harvesting is up to me						
0.35 (−0.07, 0.78) *p* = 0.11	Hoonah Juneau Sitka	0.45 (−0.12, 1.02) 0.42 (−0.48, 1.31) 0.09 (−0.68, 0.86) *p* = 0.79	Alaska Native Non‐Native	0.42 (−0.11, 0.95) 0.25 (−0.43, 0.93) *p* = 0.70	Boy Girl Nonbinary	0.48 (−0.14, 1.11) 0.17 (−0.49, 0.84) 0.43 (−0.71, 1.57) *p* = 0.79
Q15 (Behavioral intention): I intend to check the SEATOR website to help decide if I should go clam harvesting						
0.07 (−0.41, 0.54) *p* = 0.78	Hoonah Juneau Sitka	0.05 (−0.56, 0.66) 0.14 (−0.90, 1.19) 0.00 (−0.83, 83) *p* = 0.97	Alaska Native Non‐Native	0.28 (−0.27, 0.84) −0.23 (−0.98, 0.52) *p* = 0.27	Boy Girl Nonbinary	−0.12 (−0.82, 0.57) 0.22 (−0.47, 0.91) 0.28 (−1.01, 1.57) *p* = 0.75
Model 1: Overall association: Tests for the association between pre‐ and post‐program response means (i.e., the intervention effect), adjusting for site, age, Alaska Native identity, and gender identity						
Model 2: Effect modification by geography: Tests for heterogeneity in the association between pre‐ and post‐program response means by site, adjusting for age, Alaska Native identity, and gender identity						
Model 3: Effect modification by Alaska Native identity: Tests for heterogeneity in the association between pre‐ and post‐program response means by Alaska Native identity, adjusting for site, age, and gender identity						
Model 4: Effect modification by gender identity: Tests for heterogeneity in the association between pre‐ and post‐program response means by gender identity, adjusting for site, age, and Alaska Native identity						

## Appendix D Individual Participants' Post‐Program Survey Responses Related to Behaviors and Behavioral Intentions Compared to 1 Year Post‐Program Response. Likert‐Scale Responses, Ranging From Disagree (1) to Agree (5)

**Table:**

	Q7. I have participated in subsistence clam harvesting		Q8. Since learning about clam harvest safety, I feel more willing to participate in subsistence clam harvesting		Q15. I intend to check the SEATOR website to help decide if I should go clam harvesting		Q16. Since I have found out about the resources available on the SEATOR website, I have used them when deciding whether to clam harvest	
	Post‐program	One year post program	Post‐program	One year post program	Post‐program	One year post program	Post‐program	One year post program
Participant 1	5	5	5	5	5	5	5	5
Participant 2	5	5	5	5	2	5	1	5
Participant 3	4	5	5	5	5	5	5	1
Participant 4	3	5	4	4	4	5	3	3

## Appendix E Pre‐ and Post‐Program Structured Interview Script

Question 1 How often do you go clam harvesting, if ever? Who do you go with? Do you go for any particular occasion?

Question 2 In your own words, describe the process you go through before, during, and after you harvest clams.

Question 3 In your own words, how do you feel about clam harvesting? Do you enjoy it? Is it an activity you look forward to? Why or why not? Is there anything you don't like about it?

Question 4 How do the people in your life (friends, family, etc.) feel about clam harvesting? What do you think of their feelings toward clam harvesting? Have these individuals' feelings about clam harvesting influenced how you feel about harvesting? If so, how?

Question 5 How safe do you feel about harvesting and eating shellfish or clams? What makes you feel this way?

Question 6 Do you think it is possible to reduce your risk of exposure to paralytic shellfish toxins? How so? Where did you hear about these strategies to reduce exposure risk?

Question 7 Do you plan to check the SEATOR website for information on toxin levels/ warnings before you go on a shellfish harvest? Why or why not? Is there anything that might make it easier for you to check toxin levels?

Question 8 Is there anything you wish you knew more about when it comes to clam harvesting and related toxins?

Question 9 Do you believe it is possible for you to lower your risk of exposure to paralytic shellfish toxins when clam harvesting?

Question 10 Do you think your feelings about paralytic shellfish toxins make you more or less likely to check toxin levels?
